# Occupational, academic, and personal determinants of wellbeing and psychological distress in residents: results of a survey in Lyon, France

**DOI:** 10.3389/fpsyg.2024.1347513

**Published:** 2024-05-06

**Authors:** Ludivine Nohales, Emmanuel Fort, Sophie Pelloux, Clio Coste, Pierre Leblanc, Julia De Ternay, Martine Wallon, Benjamin Rolland, Jean-Baptiste Fassier, BASIL Study Group, J Haesebaert

**Affiliations:** ^1^Occupational Health and Medicine Department-CRPPE Hospices Civils de Lyon, Lyon, France; ^2^Univ Lyon, University Lyon 1 Transport Work and Environmental Epidemiology Research and Surveillance Unit – UMRESTTE (UMR T9405), Lyon, France; ^3^Service de Santé Universitaire, University Claude Bernard Lyon, Lyon, France; ^4^University Claude Bernard Lyon 1, Lyon, France; ^5^Department of Radiotherapy, Léon Bérard Cancer Center, Lyon, France; ^6^Direction Qualité Usagers et Santé Populationnelle, Hospices Civils de Lyon, Lyon, France; ^7^Research On Healthcare Performance (RESHAPE), Université Claude Bernard Lyon INSERM U1290, Lyon, France; ^8^SUAL, Hospices Civils de Lyon, Bron, France; ^9^Institut des Agents Infectieux, Hospices Civils de Lyon, Lyon, France; ^10^Waking Team, CRNL University Claude Bernard Lyon, Bron, France; ^11^SUAL Le Vinatier, Hospices Civils de Lyon, Bron, France; ^12^Inserm U1028, CNRS UMR5292, University Claude Bernard Lyon 1, Bron, France

**Keywords:** academic conditions, job strain, mental health, psychological distress, resident, wellbeing

## Abstract

**Introduction:**

The mental health of residents is a growing significant concern, particularly with respect to hospital and university training conditions. Our goal was to assess the professional, academic, and psychological determinants of the mental health status of all residents of the academy of Lyon, France.

**Materials and methods:**

The Health Barometer of Lyon Subdivision Residents (BASIL) is an initiative which consists in proposing a recurrent online survey to all residents in medicine, pharmacy, and dentistry, belonging to the Lyon subdivision. The first of these surveys was conducted from May to July 2022. Participants should complete a series of validated questionnaires, including the Warwick-Edinburgh Mental Wellbeing Scale (WEMWBS), and the Kessler Psychological Distress Scale (K6), respectively, and *ad-hoc* questions assessing their global health and hospital and academic working conditions. A Directed Acyclic Graph (DAG) analysis was conducted prior to multivariable analyses, to explore the determinants associated with low wellbeing (WEMWBS <43) and high psychological distress (K6 ≥ 13).

**Results:**

A total of 904 residents (response rate: 46.7%) participated in the survey. A low level of wellbeing was observed in 23% of participants, and was significantly associated to job strain (OR = 2.18; 95%CI = [1.32–3.60]), low social support (OR = 3.13; 95%CI = [2.05–4.78]) and the experience of very poor university teaching (OR = 2.51; 95%CI = [1.29–4.91]). A high level of psychological distress was identified for 13% of participants, and associated with low social support (OR = 2.41; 95%CI = [1.48–3.93]) and the experience of very poor university teaching (OR = 2.89, 95%CI = [1.16–7.21]).

**Conclusion:**

Hospital working conditions, social support, and the perception of teaching quality, were three major determinants of wellbeing and psychological distress among health profession residents. Demographic determinants, personal life and lifestyle habits were also associated. This supports a multilevel action in prevention programs aiming to enhance wellbeing and reduce mental distress in this specific population and local organizational specificities.

## 1 Introduction

The overall state of health of healthcare professionals, including mental health is a growing major concern at the international level. According to the World Health Organization (WHO), “mental health is a state of mental wellbeing that enables people to cope with the stresses of life, realize their abilities, learn well and work well, and contribute to their community.” It lies on a complex continuum, with widely varying levels of difficulty and distress. From a bio-psycho-social perspective, mental health stems from individual, social and structural determinants. Individual biological and psychological factors, such as emotional competence or personality traits, and exposure to adverse circumstances (social, academic, professional, political, economic, environment etc.), contribute to the risk of poor mental health. Protective factors are resources to be supported throughout life, such as individual social and emotional skills, social support, decent work, community cohesion, etc. (Mental Health, World Health Organization, 2022).

According to the Big Five model, the five major personality traits (neuroticism, conscientiousness, extraversion, agreeableness and openness to experience) are involved in the way individuals behave and interact with their environment, in their personal and professional relationships, in life experiences. They also influence the way individuals make choices and transform their environment (McCrae and John, 1992). They influence mental health along with stress exposure and coping strategies. In complex work environments such as hospitals, high levels of neuroticism and conscientiousness could be linked to poorer mental health, in contrast to extraversion, openness to experience and agreeableness, especially when those two traits are combined (Doherty and Nugent, 2011; Delafontaine et al., 2024).

Risks and protective factors of mental health are found in society at different levels, for example in the workplace. According to the Job Strain model, poor working conditions result from an unbalanced relationship between job control (high/low) and psychological job demand (high/low), and are associated with negative effects on occupational stress and health, including mental health (low wellbeing, psychological distress, anxiety, etc.). A high level of occupational stress is associated with high job demand and low job control, known as “job strain.” If little support from superiors and/or colleagues is added, job stress is increased (Karasek, 1979; Niedhammer, 2002; Laditka et al., 2023).

Thus, the impact of psycho-social risk factors, workplace stress and violence, has been identified as crucial determinants of psychological wellbeing (Rugulies et al., 2023) in particular in the healthcare sector (Hu et al., 2019; Kansoun et al., 2019; Dres et al., 2023). The overall proportion of healthcare workers who report experiencing violence at work has been estimated to reach nearly 80% (Rossi et al., 2023). While all healthcare professionals can be affected by stress and violence, young healthcare professionals display specific vulnerability features. Residents in medicine, pharmacy, and dentistry, have a dual status as both students and caregivers, and they may experience specific, cumulative, and recurring stress factors. Their working conditions in hospital involve heavy workloads, emotionally demanding situations, and high professional expectations (Mara, 2018). They are in the process of constructing their knowledge, their experience and professional identity within a competitive work environment and an uncertain professional future. From this perspective, academic conditions are essential. Their status as university students requires them to take and validate training courses (theoretical knowledge), and follow a hospital residency program (experiential knowledge). The imperative to continue their residency course may impede their ability to extricate themselves from potentially harmful situations.

The combination of these factors can have adverse consequences on their mental health (Papp et al., 2004; Sen et al., 2010; Ogawa et al., 2018; Zhou et al., 2020; Awan et al., 2022). International reviews suggest that the prevalence of burnout among medical residents ranges from 35.7% (Rodrigues et al., 2018) to 51% (Low et al., 2019), and depression or depressive symptoms from 20.9% to 43.2 (Mata et al., 2015), respectively. Moreover, it has been consistently found a more degraded sense of personal accomplishment and depersonalization among residents, compared to senior doctors (Kansoun et al., 2019). In France, the exact annual number of work-related suicides among residents is poorly known due to the lack of any reporting obligation, families’ desire for discretion, and the lack of knowledge regarding the exact cause of death, particularly the causality of working conditions (Santé Publique France, 2021). Another important point is that the mental health of residents was particularly impacted by the coronavirus pandemic in 2019. Two French national studies found a deterioration in the level of psychological distress (high for 83% of students, compared with 21% initially), with a consumption of psychotropic medications which has more than doubled (Frajerman et al., 2022; Rolland et al., 2022). Previous studies have highlighted the use of psychoactive substances by individuals, including health professionals, as a strategy to cope with difficult working conditions, regulate stress and reduce unpleasant emotions. As young caregivers, residents could present higher levels of psychological distress, and thus, be more at risk of substance use, as poor mental health has been associated, for instance, with increased alcohol consumption (Lebensohn et al., 2013; Stimpfel et al., 2020).

The consequences of poor mental health among residents are substantial for themselves and the healthcare system (e.g., interrupted studies), but also for patients, as they lead to risks for the quality and safety of care, including the risk of medical errors (Dewa et al., 2014, 2017; Pereira-Lima et al., 2019; Frajerman, 2020a; Mangory et al., 2021; Hodkinson et al., 2022). A medical error may have a deep and lasting emotional impact, may affect the ability to provide patient care, can lead to medical malpractice lawsuits and increased healthcare costs (Burlison et al., 2017; Robertson and Long, 2018; Rodziewicz et al., 2024).

More generally, the impact of residents’ occupational and training conditions on their overall health is a growing concern, and action is needed to promote their mental health at work (Lietor et al., 2021). In this perspective, the University of Claude Bernard Lyon 1 (UCBL) and the University Hospital of Lyon have developed an action plan based on a socio-ecological model (Wold and Mittelmark, 2018; Frajerman, 2020b) to enhance the mental health, training and working conditions of residents in the Lyon subdivision. A collaborative approach to health program planning, inspired by the intervention mapping protocol (Bartholomew et al., 2016), has been implemented by University Health Unit (« SSU ») and the hospital Occupational Medicine Unit (« SMST »). The first step of this protocol involved a recurrent assessment of residents’ health condition, aiming to guide the decisions on which actions should be implemented (Mara, 2018). The Health Barometer of Lyon Subdivision Residents (« Baromètre Santé des Internes de la Subdivision de Lyon », BASIL) is a recurrent survey created to serve as a recurrent “diagnostic” step. To date, only one survey was conducted in 2022, while the next one is planned in 2024.

The primary objective of the 2022 BASIL survey was to assess the mental health of health profession residents of the Lyon subdivision. The secondary objectives were to explore the relationships between their mental health, and their working and academic conditions, taking into account lifestyle habits, and personality traits.

## 2 Methods

### 2.1 Study design

BASIL is an online survey, using both validated self-reported questionnaires and *ad hoc* questions. Here, only the data of the 2022 edition were used.

### 2.2 Study population and data collection methods

The survey was offered to all registered residents in the Lyon subdivision (*n* = 1,936), encompassing those in medicine, pharmacy and dentistry. Residents were invited to participate by an email sent on their personal address provided by the school departments. The email contained explanations on the study and the link to the survey (unique URL). Three reminders were sent on June 15th, June 27th, and July 4th, 2022, respectively. The survey was also relayed on social networks by Lyon resident unions, and on the Lyon University hospital and Claude Bernard Lyon 1 University intranet sites. Data were collected through an online survey on the Lime survey platform that was accessible from May 30 to July 15, 2022.

### 2.3 Assessment tools

The study questionnaire was constructed by a participatory working group, including residents and their representatives (unions), as well as representatives from hospital and university management, and researchers from various disciplines (i.e., occupational medicine, mental health and addiction medicine, university student health service, public health, and sleep medicine).

The variables collected were:

- socio-demographic: sex, age, familial status.
- academic: year of residency, specialty, evaluation of the postgraduate university courses, satisfaction with the choice of specialty, wish of an academic career.
- occupational: working conditions, work-time characteristics, work-organization constraints, violence at work.
- lifestyle: food, sleep, sport, drug use.

The following validated self-reported questionnaires were used:

- Wellbeing: Warwick-Edinburgh Mental Wellbeing Scale (WEMWBS) (Tennant et al., 2007; Trousselard et al., 2016): The WEMWBS is a 14-item scale validated in French, designed to assess psychological wellbeing in adults by exploring both hedonic (absence of suffering and pleasure) and eudemonic (sense of self and accomplishment) dimensions. The total score ranges from 14 to 70 with a score below 43 indicating low psychological wellbeing (according to the excel spreadsheet for calculating scores with WEMWBS sent after completing the registration for a License to use WEMWBS for non-commercial purposes).
- Working conditions: The Job Content Questionnaire (Niedhammer, 2002; Niedhammer et al., 2006) is an international questionnaire validated in French, based on the “Karasek” model and evaluating psychological demand, decision latitude and social support at work. It comprises 26 items on a 4-point Likert scale. Thresholds are set at 21 for psychological demand, 70 for decision latitude and 23.9 for social support. The combination of psychological demand and decision latitude scores defines the four work situations (“passive,” “active,” “relaxed” and “stressed”).
- Personality traits: Big Five Inventory BFI-10 French items (Plaisant, 2010; Courtois et al., 2020): this concise self-reported questionnaire consists of 10 items extracted from the 44-item BFI, deemed the most representative of the various facets.
- Psychological distress: Kessler Psychological Distress Scale (K6) (Kessler et al., 2003; Gouvernement du Canada, 2016): the K6 is a self-reported questionnaire whose items are classified on a five-point scale, with a highest score of 24. A score ≥13 indicates a high probability of severe psychological distress, while a score < 13 indicates that severe psychological distress is unlikely.
- Alcohol consumption: AUDIT-C (Bush et al., 1998) is a condensed version comprising the first 3 items of AUDIT (10-item scale). The three items of this self-reported questionnaire use a 4-point scale with a maximum score of 12 points.

According to epidemiological models (Stoltzfus, 2011), the variables of interest for mental health were categorized in two groups for both the wellbeing and the psychological distress. The first outcome was the low wellbeing which was defined by having a WEMWBS score below 43. The second outcome was the high level of psychological distress which was defined by having a K6 score equal or greater than 13.

The entire questionnaire is available in English in Supplementary material.

### 2.4 Statistical analysis

Compared with data from the university’s education department, preliminary results indicated an over-representation of women, and a different distribution across age groups and specialties. Consequently, a margin calibration was conducted (Sautory, 1993; INSEE, 2021). The SAS CALMAR macro (“CALage sur MARges”) is used to adjust a sample from a sample survey, by re-weighting individuals, utilizing auxiliary information available on several variables, referred to as calibration variables.

The chosen calibration method was margin calibration - logit method (initial weights between zero and three). The decision was made to incorporate gender, age groups (6 categories) and specialties (grouped into 12 categories). This method yielded the least dispersion in calibration weights. Thus, the margin calibration method ensure the representativeness of the sample.

Descriptive statistics are presented as frequency, percentage and 95% confidence interval (95%CI) for categorical variables, and as mean and 95% confidence interval for continuous variables.

Descriptive statistical analyses were conducted using the surveyfreg and surveymeans (Koch et al., 1975; Bedrick, 1983) procedures of SAS software version 9.4 account for these sampling weights.

### 2.5 Directed acyclic graph

Mental health status was assessed independently, based on both the WEMWBS (wellbeing) and K6 (psychological distress) questionnaire. To assess the association between working conditions, academic conditions and the mental health of residents, a Directed Acyclic Graph (DAG) analysis was conducted prior to the analyses (Digitale et al., 2022). DAGs serve as a method to address causal questions in clinical and epidemiologic research, guiding study design and statistical analysis. DAGs are constructed to illustrate existing knowledge about biological and behavioral systems relevant to specific causal research questions. Through this, DAGs can identify variables sufficient to eliminate confounding and some forms of selection bias.

The authors developed four DAGs (wellbeing/working conditions; wellbeing/academic conditions; psychological distress/working conditions; psychological distress/academic conditions) (Supplementary material). The variables required for fitting the regression models were defined using the free DAGitty software, accessible at https://www.dagitty.net/.

The adjustment variables for investigating the connection between low wellbeing (Model 1 = M1) or a high probability of severe psychological distress (Model 2 = M2), and the main occupational explanatory variables (low social support and the quadrants of the Job content Questionnaire) were age, year of residency, specialty, and four dimensions of the BFI (Extraversion, Conscientiousness, Neuroticism, Openness).

The adjustment variables for examining the association between low wellbeing (Model 3 = M3) or a high probability of severe psychological distress (Model 4 = M4), and academic conditions (Evaluation of the contribution of the postgraduate teaching to the training, frequency of weekly half a day of university training, frequency of weekly half a day of personal training, tutor) were age, year of residency, specialty, and four dimensions of the BFI (Extraversion, Conscientiousness, Neuroticism, Openness).

We performed separate models for working conditions and academic conditions because we wanted to independently assess the effects of working conditions and academic conditions, for both low wellbeing and high psychological distress. For each model, we performed multivariable logistic regression based on the variables required for fitting the regression models according to each of the four DAG. Analyses were carried out using weighted logistic regressions (Cassel, 2006). The SAS “surveylogistic” procedure was employed to consider the sample design (Koch et al., 1975; Bedrick, 1983). Results were presented as weighted odd ratio (OR) and 95% confidence interval (95%CI).

### 2.6 Research ethics

The BASIL survey protocol was agreed on April 14, 2022, by the University of Lyon’s research ethics committee (CER-UdL n° 2022-03-17-002).

A CNIL declaration was made through the University of Claude Bernard Lyon 1’s data protection officer on March 18, 2022 (n°2022–006).

An information notice was attached to the e-mail inviting participation in the survey. Participation in the survey was anonymous and voluntary. No identifying data was collected.

**Figure 1:**
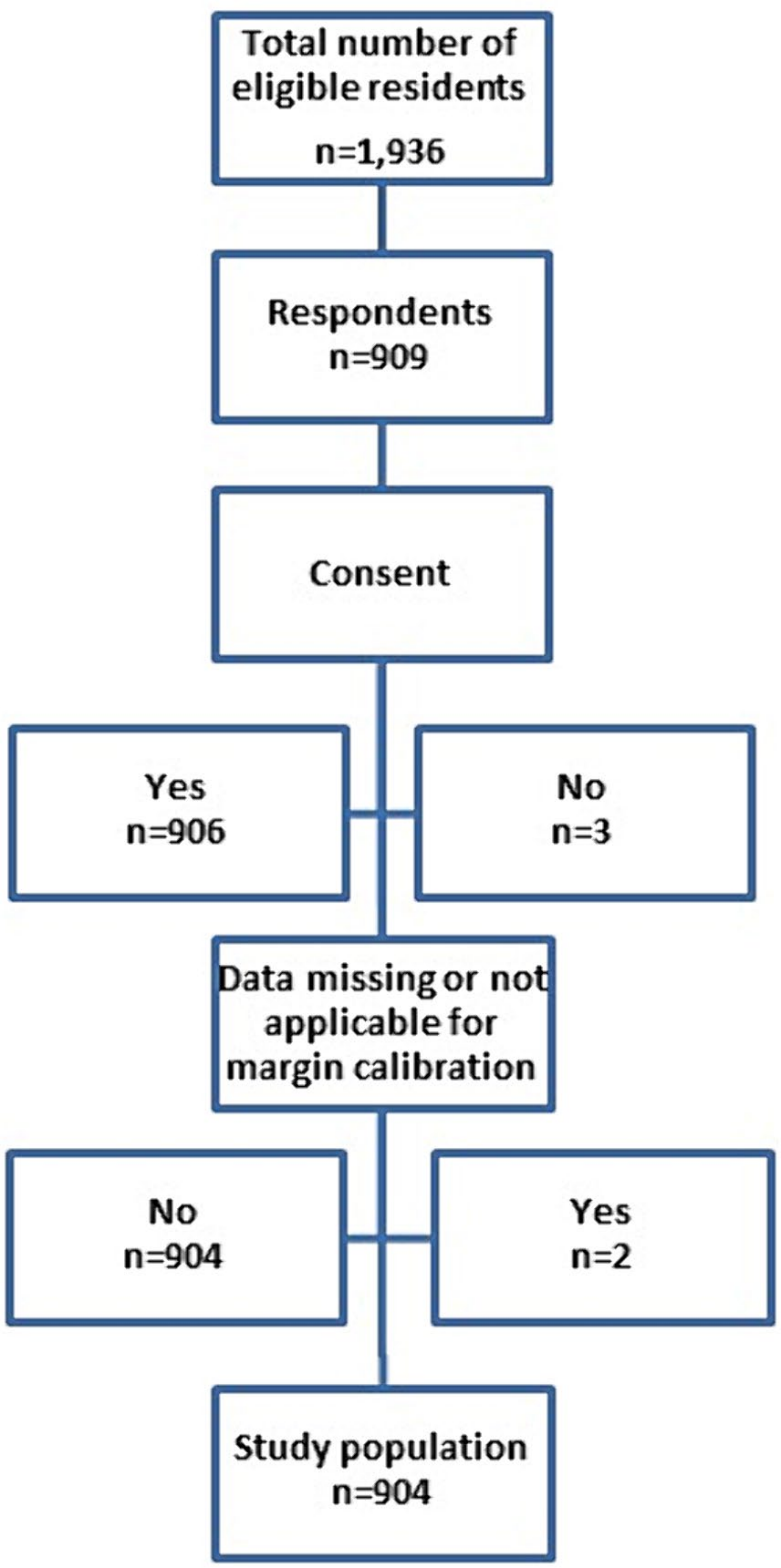
Flow chart of the BASIL study.

At the start of the online questionnaire, a message explained participants’ rights and collected their consent to participate, with a box that had to be ticked in order to access the rest of the BASIL questionnaire (Figure 1).

## 3 Results

### 3.1 Study population

Of the 1,936 registered residents in the Lyon subdivision, a total of 904 residents completed the entire questionnaire (participation rate: 46.7%) (Figure 1). Residents in medical studies (90.1%), pharmacy (8.6%) and dentistry (1.3%), were categorized into eight classes of specialties. Among medical residents, 101 (12.5%) were in Anaesthesia-intensive care or Emergency and critical care, 234 (24.6%) in General medicine, 50 (4.1%) in Pediatrics, 56 (8.6%) in Pharmacy (excluding medical biology), 51 (5.6%) in Psychiatry, 61 (6.5%) in Radiology or Nuclear Medicine or Genetics or Anatomopathology or Medical Biology, 121 (16.0%) in other surgical specialties or dentistry or Gynecology-Obstetrics and 230 (22.2%) in other medical specialties or Occupational medicine or Public health (Table 1).

**Table 1:** Description of study population.

	n (*N* = 904)	Weighted percentage	95% CI
Socio-demographic			
**Gender**			
Male	309	42.2	
Female	595	57.8	
**Age group**			
Under 24	78	13.7	
Age 25	136	19.2	
Age 26	193	21.7	
Age 27	185	18.1	
28 years old	149	12.8	
Over 29	163	14.7	
**Marital status**			
Single, divorced	298	34.3	31.0–37.6
Couple, civil union, married	606	65.7	62.4–69.0
**Child**			
No children	860	96.0	94.7–97.2
Child(ren)	44	4.0	2.8–5.3
**Living alone**			
No	632	69.7	66.5–72.9
Yes	272	30.3	27.1–33.5
**Place of the 2nd cycle student in Lyon**			
No	537	59.9	56.5–63.3
Yes	367	40.1	36.7–43.5
**Year of residency**			
1 y	203	29.0	25.6–32.4
2 y	221	25.3	22.3–28.3
3 y	232	22.7	19.9–25.4
4 y	163	15.0	12.7–17.3
≥5 y	85	8.0	6.3–9.7
**Specialty 3 groups**			
Odontology	10	1.3	0.5–2.1
Pharmacy	56	8.6	6.4–10.8
Medicine	838	90.1	87.8–92.4
Work and studies			
**Satisfied with the choice of specialty**			
Not at all	7	0.5	0–1.1
Moderately	108	11.8	9.6–14.1
Completely	789	87.6	85.4–89.9
**Had chosen to change their initial specialty**			
No	864	95.4	93.3–97.1
Yes	42	4.6	2.9–5.7
**Wish of an academic career**			
Do not know	178	21.5	18.5–24.4
No	647	69.3	66.0–72.6
Yes	79	9.2	7.1–11.3
**Work more than 48 h weekly**			
No	371	41.1	37.7–44.6
Yes	531	58.9	55.4–62.3
**Shift work**			
No	252	28.1	24.9–31.2
Yes	652	71.9	68.8–75.1
**Free to dispose of the weekly half-day of university training**			
Always	228	26.3	23.2–29.4
Almost always	194	21.9	18.9–24.8
Sometimes	177	18.9	16.2–21.6
Rarely	132	13.5	11.2–15.8
Never	173	19.4	16.6–22.2
**Free to dispose of the weekly half-day of personal training**			
Always	174	20.0	17.2–22.8
Almost always	142	16.5	13.9–19.2
Sometimes	129	14.1	11.7–16.5
Rarely	181	18.9	16.2–21.6
Never	278	30.4	27.2–33.6
**Rate the contribution of the postgraduate teaching to training**			
Very poor	160	18.0	15.3–20.6
Poor	310	33.4	30.1–36.6
Fairly good	238	26.2	23.2–29.3
Good	160	18.4	15.6–21.1
very good	36	4.1	2.7–5.5
**Become again what they have chosen**			
Definitely not	45	4.5	3.1–5.8
Probably not	116	12.6	10.3–14.9
Not sure	188	19.8	17.1–22.6
Probably yes	288	32.5	29.2–35.8
Definitely yes	267	30.6	27.4–33.9
**Have already been a victim of violence (verbal, physical, psychological, or sexual)**			
No	598	67.9	64.7–71.2
Yes	306	32.1	28.8–35.3
Health			
**At present, how would you rate your general state of health?**			
Very good	178	21.1	18.2–24
Good	441	47.8	44.4–51.3
Neither good nor poor	196	21.5	18.7–24.4
Poor	83	9.0	7.0–10.9
Very poor	6	0.6	0.1–1.1
**Have enough time to eat on the days when they are on placement**			
Never	35	3.9	2.5–5.2
Rarely	129	14.7	12.2–17.1
Sometimes	195	21.1	18.3–23.9
Often	336	36.6	33.3–40
Very often	209	23.8	20.8–26.8
	n	Weighted mean	95% CI
Time for moderate physical activity per week (hours) (e.g., walking)	904	3.0	2.8–3.2
	n	Weighted mean	95% CI
Time spent sitting per day (hours) (considering all situations: work, transport, home, leisure…)	904	7.3	7.1–7.5
**Sleep difficulties interfere with the daily functioning**			
Not at all	233	25.5	22.4–28.5
Slightly	312	34.6	31.2–37.9
Moderately	197	22.3	19.3–25.3
Very	132	14.4	12.0–16.8
Extremely	30	3.3	2.1–4.5
**Professional screen exposure**			
Less than 4 h included	168	20.3	17.4–23.2
Between 4 and 6 h	113	12.2	10.0–14.4
Between 6 h and 8 h	303	32.9	29.6–36.1
More than 8 h	320	34.6	31.3–38
**Smoking status**			
Never smoked	599	66.1	62.8–69.4
I used to smoke, but I do not anymore	91	9.7	7.7–11.8
I smoke occasionally (not every day)	136	15.7	13.1–18.2
I smoke tobacco daily (at least 1 cigarette a day)	78	8.5	6.6–10.4
**Cannabis use in the last 30 days**			
Never	815	89.7	87.6–91.9
1–2 times	60	6.8	5.1–8.6
3–9 times	23	2.7	1.5–3.8
10 times or more	4	0.4	0–0.9
Every day	2	0.3	0–0.8
	n	Weighted mean	95% CI
**AUDIT-C score (0–12)**			
Overall	904	3.2	3.1–3.3
Female	595	3.0	2.8–3.1
Male	309	3.5	3.3–3.8

The majority of participants were female (*n* = 595, 57.8%), lived with a partner (*n* = 606, 65.7%), and had no children (*n* = 860, 96.0%). Approximately 30.3% of residents (*n* = 272) reported living alone. The average age of respondents was 27 years (*SD* = 2.2 years).

Most residents had studied outside the Lyon subdivision (*n* = 537, 59.9%). Participants were proportionally more numerous in the first (*n* = 203, 29.0%) and second year of residency (*n* = 221, 25.3%).

### 3.2 Working and study conditions

In total, 58.9% of respondents (*n* = 531) reported working more than 48 h, with an average weekly workload of 51.2 h, including on-call duty. Additionally, over 3.2% reported working 80 h or more per week. Nearly 10% of residents expressed a desire to pursue an academic career (*n* = 79, 9.2%) (Table 1).

The majority of residents were satisfied with the choice of their specialty (*n* = 789, 87.6%), and 4.3% (*n* = 42) had previously opted to change their initial specialty.

Among interviewees, 68.9% rated their overall health during the survey period as good or very good (*n* = 619, 68.9%). Residents reported sitting for an average of 7.3 h a day. They reported being engaged in 2 h of intense physical activity per week and 3 h of moderate activity. Unsatisfactory sleep disrupted daily life for 17.7% of residents (*n* = 162). Only 60.4% (*n* = 545) of residents often/very often had time to eat at work. Over a half of residents (*n* = 488, 54.5%) had a positive AUDIT-C score. Anxiolytics and hypnotics had been taken more than once a month during the past 12 months for 10.3% (*n* = 100) and 6.7% (*n* = 56) of the residents, respectively.

More than a third of residents (*n* = 306, 32.1%) reported having experienced at least one form of violence during their studies. Last, 17.1% (*n* = 161) of the respondents reported that they would not choose to be a resident a second time if they had to make that choice again.

### 3.3 Level of wellbeing and related determinants

A low level of wellbeing (WEMWBS <43) was found in 23% of respondents. The outcomes of the multivariable analysis of wellbeing and working conditions in residency are presented in Table 2.

**Table 2:** Multivariable analysis by level of wellbeing and working conditions in residency.

	WEMWBS low wellbeing (score < 43) (23%)		WEMWBS moderate or high wellbeing (score ≥43) (77%)				
	n	Weighted %	n	Weighted %	OR	95% CI	*p*-value
**Age group**							<0.01
Under 24	15	11.6	63	14.3	1.00	–	
Age 25	28	16.9	108	19.8	1.24	0.54–2.84	
Age 26	38	17.9	155	22.8	1.29	0.57–2.94	
Age 27	51	20.6	134	17.3	2.85	1.14–7.11	
28 years old	33	12.5	116	12.8	2.18	0.85–5.58	
Over 29 years old	51	20.5	112	12.9	5.07	2.03–12.66	
**Year of residency**							<0.05
1 y	48	28.3	155	29.2	1.00	-	
2 y	58	27.2	163	24.7	0.67	0.36–1.25	
3 y	48	19.8	184	23.5	0.44	0.22–0.88	
4 y	47	18.9	116	13.9	0.44	0.20–0.95	
≥5 y	15	5.8	70	8.7	0.18	0.07–0.48	
**Specialties**							NS
Anaesthesia-intensive care/Emergency and critical care	24	13.1	77	12.4	1.51	0.76–3.01	
General medicine	50	21.1	184	25.6	1.00	-	
Pediatrics	16	5.75	34	3.6	0.91	0.44–1.90	
Pharmacy (excluding medical biology)	14	8.3	42	8.7	1.34	0.76–2.37	
Psychiatry	11	4.8	40	5.8	1.21	0.52–2.83	
Radiology/Nuclear Medicine/Genetics/Anatomopathology/Medical Biology	14	6.7	47	6.4	1.51	0.64–3.53	
Other surgical specialties/Odontology/Gynecology-Obstetrics	27	15.7	94	16.0	1.80	0.75–4.32	
Other medical specialties/Occupational medicine/Public health	60	24.5	170	21.5	1.50	0.67–3.34	
**Social support at work**							<0.001
High (>23.9)	105	49.3	548	79.9	1.00	-	
Low (≤23.9)	111	50.7	140	20.1	3.13	2.05–4.78	
**Quadrants of Job Content Questionnaire**							<0.001
Relaxed work (low psychological demand, high decision latitude)	21	9.8	247	35.4	0.34	0.18–0.66	
Passive work (low psychological demand, low decision latitude)	44	20.0	119	17.6	1.10	0.63–1.98	
Active work (high psychological demand, high decision latitude)	48	22.3	177	25.9	1.00	-	
Stressed work (high psychological demand, low decision latitude)	103	47.9	145	21.07	2.18	1.32–3.60	
**Extraversion**	n	216	688		1.00	-	<0.001
	Mean (SD)	2.89 (0.09)	3.27 (0.05)		0.52	0.42–0.65	
**Conscientiousness**	n	216	688		1.00	-	<0.01
	Mean (SD)	2.05 (0.06)	1.89 (0.03)		1.42	1.11–1.80	
							
**Neuroticism**	n	216	688		1.00	-	<0.001
	Mean (SD)	3.31 (0.06)	3.15 (0.04)		2.06	1.47–2.89	
**Openness**	n	216	688		1.00	-	NS
	Mean (SD)	2.72 (0.08)	2.63 (0.04)		1.05	0.85–1.29	

NS, Not significant.

The results of the multivariable analysis of wellbeing and academic conditions in residency are presented in Table 3.

**Table 3:** Multivariable analysis by wellbeing and academic conditions.

	WEMWBS low wellbeing (score < 43) (23%)		WEMWBS moderate or high wellbeing (score ≥43) (77%)				
	n	Weighted %	n	Weighted %	OR	95% CI	*p*-value
**Age group**							<0.001
Under 24	15	11.6	63	14.3	1.00	-	
Age 25	28	16.9	108	19.8	1.29	0.58–2.91	
Age 26	38	17.9	155	22.8	1.33	0.60–2.96	
Age 27	51	20.6	134	17.3	3.06	1.30–7.20	
28 years old	33	12.5	116	12.8	2.45	1.00–5.97	
Over 29 years old	51	20.5	112	12.9	5.33	2.24–12.67	
**Year of residency**							<0.01
1 y	48	28.3	155	29.2	1.00	-	
2 y	58	27.2	163	24.7	0.76	0.41–1.39	
3 y	48	19.8	184	23.5	0.49	0.25–0.96	
4 y	47	18.9	116	13.9	0.52	0.25–1.09	
5 y and over	15	5.8	70	8.7	0.16	0.06–0.41	
**Specialties**							NS
Anaesthesia-intensive care/Emergency and critical care	24	13.1	77	12.4	1.31	0.57–2.99	
General medicine	50	21.1	184	25.6	1.00	-	
Pediatrics	16	5.75	34	3.6	0.55	0.22–1.36	
Pharmacy (excluding medical biology)	14	8.3	42	8.7	0.76	0.55–2.85	
Psychiatry	11	4.8	40	5.8	0.78	0.29–2.07	
Radiology/Nuclear Medicine/Genetics/Anatomopathology/Medical Biology	14	6.7	47	6.4	0.67	0.24–1.92	
Other surgical specialties/Odontology/Gynecology-Obstetrics	27	15.7	94	16.0	0.98	0.35–2.79	
Other medical specialties/Occupational Medicine/Public Health	60	24.5	170	21.5	1.18	0.46–3.03	
**Free to dispose of the half-day of university training every week**							NS
Always	34	16.5	194	29.2	1.00	-	
Almost always	40	19.5	154	22.6	0.92	0.41–2.09	
Sometimes	47	21.9	130	18.0	1.44	0.63–3.26	
Rarely	41	16.3	91	12.7	1.43	0.57–3.59	
Never	54	25.7	119	17.5	1.48	0.58–3.75	
**Free to dispose of the weekly half-day of personal training**							NS
Always	24	11.2	150	22.6	1.00	-	
Almost always	29	14.7	113	17.1	1.64	0.63–4.29	
Sometimes	24	12.0	105	14.8	1.41	0.51–3.890	
Rarely	49	21.6	132	18.1	2.10	0.76–5.79	
Never	90	40.5	188	27.4	2.57	0.95–6.93	
**Evaluation of the contribution of the postgraduate teaching to training**							<0.05
Very poor	48	22.2	112	16.7	2.51	1.29–4.91	
Poor	74	34.2	236	33.2	1.50	0.79–2.85	
Fairly good	60	29.3	178	25.3	1.61	0.89–2.92	
Good	28	11.7	132	20.3	1.00	-	
Very good	6	2.6	30	4.5	0.81	0.27–2.39	
Tutor							NS
No	145	69.4	409	60.5	1.00	-	
Yes	71	30.6	279	39.4	0.58	0.31–1.08	
**Extraversion**	n	216	688		1.00	-	<0.001
	Mean (SD)	2.89 (0.09)	3.27 (0.05)		0.51	0.41–0.63	
**Conscientiousness**	n	216	688		1.00	-	<0.001
	Mean (SD)	2.05 (0.06)	1.89 (0.03)		1.53	1.22–1.92	
**Neuroticism**	n	216	688		1.00	-	<0.001
	Mean (SD)	3.31 (0.06)	3.15 (0.04)		2.20	1.60–3.03	
**Openness**	n	216	688		1.00	-	NS
	Mean (SD)	2.72 (0.08)	2.63 (0.04)		1.01	0.83–1.22	

NS, Not significant.

#### 3.3.1 Working conditions (model 1 = M1) and academic conditions (model 3 = M3) and low wellbeing

Exhibiting a low level of wellbeing was associated with job strain (OR = 2.18; 95%CI = [1.32–3.60]), low social support (OR = 3.13; 95%CI = [2.05–4.78]), and reporting the experience of a very poor contribution from university training (OR = 2.51; 95%CI = [1.29–4.91]). However, there was no significant association between a low level of wellbeing and specialty type, weekly half-day of university or personal training, or having a tutor.

#### 3.3.2 Personal determinants and low wellbeing

Variables associated with a low level of wellbeing included being older (for M1: *p* < 0.01 and for M3: *p* < 0.01), and simultaneously in the first part of residency (year 1 and 2) (for M1: *p* < 0.05 and for M3: *p* < 0.01). Regarding personality traits, a low level of wellbeing was positively associated with neuroticism (for M1: *p* < 0.001; for M3: *p* < 0.001), conscientiousness (for M1: *p* < 0.005; for M3: *p* < 0.01) while, in contrast, it was negatively associated with extraversion (for M1: *p* < 0.001; for M3: *p* < 0.001). There was no significant association with the openness dimension.

### 3.4 Level of psychological distress and related determinants

A high level of psychological distress (K6 ≥13) was identified in 13% of residents. The outcomes of the multivariable analysis of psychological distress and working conditions in residency are presented in Table 4.

**Table 4:** Multivariable analysis by level of psychological distress (Kessler) and working conditions in residency.

	High probability of severe psychological distress (score ≥13) *n* = 122		Unlikely severe psychological distress (score < 13) *n* = 784				
	n	Weighted %	n	Weighted %	OR	95% CI	*p*-value
**Age group**							NS
Under 24	6	8.0	72	14.5	1.00	-	
Age 25	16	17.1	120	19.5	1.38	0.47–4.04	
Age 26	24	20.1	169	21.9	1.38	0.46–4.13	
Age 27	24	19.0	161	17.9	1.94	0.56–6.74	
28 years old	21	14.9	128	12.4	1.81	0.52–6.33	
Over 29 years old	31	20.9	132	13.7	3.20	0.92–11.07	
**Year of residency**							NS
1 y	26	24.5	177	29.7	1.00	-	
2 y	30	26.0	191	25.2	0.65	0.30–1.41	
3 y	28	21.4	204	22.8	0.65	0.27–1.58	
4 y	28	20.3	135	14.2	0.70	0.25–1.91	
5 y and over	10	7.8	75	8.0	0.49	0.16–1.52	
**Specialties**							NS
Anaesthesia-intensive care/Emergency and critical care	11	9.2	90	13.0	0.57	0.25–1.31	
General medicine	35	26.7	199	24.3	1.00	-	
Pediatrics	9	6.5	41	3.7	0.67	0.31–1.43	
Pharmacy (excluding medical biology)	12	13.0	44	8.0	0.41	0.20–0.85	
Psychiatry	8	6.2	43	5.5	1.39	0.54–3.63	
Radiology/Nuclear Medicine/Genetics/Anatomopathology/Medical Biology	8	7.1	53	6.4	1.59	0.56–4.46	
Other surgical specialties/Odontology/Gynecology-Obstetrics	19	18.2	102	15.6	1.33	0.49–3.61	
Other medical specialties/Occupational medicine/Public health	20	13.1	210	23.6	0.97	0.35–2.70	
**Social support at work**							<0.001
High (>23.9)	56	47.1	597	76.7	1.00	-	
Low (≤23.9)	66	52.9	185	23.3	2.42	1.48–3.93	
**Quadrants of Job Content Questionnaire**							<0.001
Relaxed work (low psychological demand, high decision latitude)	10	6.7	258	32.9	0.17	0.08–0.39	
Passive work (low psychological demand, low decision latitude)	19	15.4	144	18.6	0.63	0.30–1.30	
Active work (high psychological demand, high decision latitude)	32	26.5	193	24.9	1.00	-	
Stressed work (high psychological demand, low decision latitude)	61	51.4	187	23.6	1.56	0.89–2.73	
**Extraversion**	n	122	782		1.00	-	NS
	Mean (SD)	3.30 (0.11)	3.17 (0.04)		0.80	0.59–1.08	
**Conscientiousness**	n	122	782		1.00	-	<0.01
	Mean (SD)	2.14 (0.08)	1.89 (0.03)		1.54	1.16–2.05	
**Neuroticism**	n	122	782		1.00	-	<0.01
	Mean (SD)	3.54 (0.07)	3.13 (0.03)		2.10	1.33–3.30	
**Openness**	n	122	782		1.00	-	NS
	Mean (SD)	2.66 (0.1)	2.65 (0.04)		1.03	0.81–1.33	

NS, not significant.

The results of the multivariable analysis of psychological distress and academic conditions in residency are presented in Table 5.

**Table 5:** Multivariate analysis by level of psychological distress (Kessler) and academic conditions.

	High probability of severe psychological distress (score ≥13) *n* = 122		Unlikely severe psychological distress (score < 13) *n* = 784				
	n	Weighted %	n	Weighted %	OR	95% CI	*p*-value
**Age group**							NS
Under 24	6	8.0	72	14.5	1	-	
Age 25	16	17.1	120	19.5	1.40	0.46–4.28	
Age 26	24	20.1	169	21.9	1.66	0.53–5.14	
Age 27	24	19.0	161	17.9	2.43	0.70–8.46	
28 years old	21	14.9	128	12.4	2.37	0.65–8.60	
Over 29 years old	31	20.9	132	13.7	4.23	1.21–14.77	
**Year of residency**							NS
1 y	26	24.5	177	29.7	1.00	-	
2 y	30	26.0	191	25.2	0.76	0.35–1.61	
3 y	28	21.4	204	22.8	0.66	0.28–1.55	
4 y	28	20.3	135	14.2	0.73	0.27–1.95	
5 y and over	10	7.8	75	8.0	0.36	0.12–1.12	
**Specialties**							NS
Anaesthesia-intensive care/Emergency and critical care	11	9.2	90	13.0	0.80	0.30–2.14	
General medicine	35	26.7	199	24.3	1.00	-	
Pediatrics	9	6.5	41	3.7	0.57	0.19–1.75	
Pharmacy (excluding medical biology)	12	13.0	44	8.0	0.34	0.11–1.07	
Psychiatry	8	6.2	43	5.5	1.32	0.40–4.40	
Radiology/Nuclear Medicine/Genetics/Anatomopathology/Medical Biology	8	7.1	53	6.4	0.80	0.21–3.03	
Other surgical specialties/Odontology/Gynecology-Obstetrics	19	18.2	102	15.6	1.33	0.35–5.01	
Other medical specialties/Occupational Medicine/Public Health	20	13.1	210	23.6	1.14	0.32–4.03	
**Free to dispose of the half-day of university training every week**							NS
Always	27	23.1	201	26.7	1.00	-	
Almost always	21	17.8	173	22.5	0.54	0.22–1.29	
Sometimes	22	17.7	155	19.1	0.72	0.30–1.73	
Rarely	23	16.6	109	13.1	0.88	0.35–2.21	
Never	29	24.7	144	18.6	0.98	0.34–2.82	
**Free to dispose of the weekly half-day of personal training**							NS
Always	19	14.7	155	20.8	1.00	-	
Almost always	18	15.7	124	16.7	2.03	0.70–5.86	
Sometimes	10	8.9	119	14.9	1.18	0.37–3.80	
Rarely	28	21.9	153	18.4	2.50	0.89–7.04	
Never	47	38.8	231	29.2	2.65	0.92–7.63	
**Evaluation of the contribution of the postgraduate teaching to training**							<0.05
Very poor	35	30.1	125	16.2	2.89	1.16–7.21	
Poor	44	36.0	266	33.0	1.69	0.73–3.87	
Fairly good	28	22.2	210	26.8	1.21	0.53–2.79	
Good	13	10.5	147	19.5	1.00	-	
Very good	2	1.2	34	4.5	0.37	0.08–1.84	
**Tutor**							NS
No	78	65.5	476	62.2	1.00	-	
Yes	44	34.5	306	37.8	0.68	0.29–1.64	
**Extraversion**	n	122	782		1.00	-	NS
	Mean (SD)	3.30 (0.11)	3.17 (0.04)		0.75	0.56–1.01	
**Conscientiousness**	n	122	782		1.00	-	<0.001
	Mean (SD)	2.14 (0.08)	1.89 (0.03)		1.61	1.22–2.12	
**Neuroticism**	n	122	782		1.00	-	<0.001
	Mean (SD)	3.54 (0.07)	3.13 (0.03)		2.25	1.49–3.39	
**Openness**	n	122	782		1.00	-	NS
	Mean (SD)	2.66 (0.1)	2.65 (0.04)		1.00	0.80–1.25	

NS, Not significant.

#### 3.4.1 Working conditions (model 2 = M2) and academic conditions (model 4 = M4) and high level of psychological distress

A high level of psychological distress (K6 ≥13) was significantly (*p* < 0.01) associated with low social support (OR = 2.41; 95%CI = [1.48–3.93]).

Low psychological distress (K6 < 13) was highly significantly (*p* < 0.001) associated with “relaxed work” (OR = 0.17; 95%CI = [0.08–0.39]). A high level of psychological distress was significantly (*p* < 0.05) associated with an increased risk of judging the contribution of university teaching as very poor (OR = 2.89; 95%CI = [1.16–7.21]).

There was no significant association between the high level of psychological distress and the type of specialty or the fact of having a half-day of weekly university training or personal training or the fact of having a tutor.

#### 3.4.2 Personal determinants and high level of psychological distress

The variables associated with a high level of psychological distress were a higher average level of neuroticism (for M2: *p* < 0.001; for M4: *p* < 0.001) and conscientiousness (for M2: *p* < 0.01; for M4: *p* < 0.01).

There was no significant association between high level of psychological distress and age, year of boarding, openness dimension or extraversion.

## 4 Discussion

### 4.1 Main findings

The main aim of the BASIL 2022 survey was to assess resident wellbeing, working and academic conditions, and personal lifestyle habits, and to explore the features associated with low wellbeing, and high level of psychological distress.

The results show that overall, most of the participants have an acceptable to very good level of wellbeing. However, other indicators give cause for concern, with a significant number of respondents reporting poor health, low levels of wellbeing and high levels of psychological distress. Multivariable analyses found that the residents’ mental health was significantly influenced by factors related to their hospital and faculty work conditions, personal life, and psychological factors associated with their personality trait. These results emphasize the need for a comprehensive approach to prevent and promote the mental health of residents, considering their working, training and personal conditions.

The WEMWBS questionnaire is not widely used among residents and thus, which makes comparisons with other studies in this specific population difficult. For instance, in 2019, Swiss general medicine residents reported a mean Wellbeing score of 51 (SD = 7.6) which is quite similar to that found in our study. Moreover, Swiss residents reported stress factors such as the lack of time for private life, heavy workload, including administrative work, and high perceived professional demands (Lindemann et al., 2019). Moreover, the prevalence of high psychological distress in BASIL is comparable to the prevalence of depressive symptoms identified in 12% of the French population aged between 25 and 34 (Hazo et al., 2022).

Regarding working conditions in the hospital residency, our survey assessed several psychosocial risk factors. A lower level of social support was associated with a poorer level of wellbeing and mental health, while the presence of support had the opposite effect. Job strain, characterized by high demand and low decision latitude, was associated with a poorer level of wellbeing. These results align with previous studies (Amiri and Behnezhad, 2020) and a recent literature review (Rugulies et al., 2023). French and European standards enforce a maximum working week of 48 h (République Française, 2015). In a previous study, it was found that working over 80 h per week increased the risk of depression among residents (Awan et al., 2022). However, in contrast with previous findings (Ogawa et al., 2018; Li et al., 2023), the BASIL study did not find a significant association between working hours and mental health status. This difference could be attributed to the fact that our study pertained to wellbeing and psychological distress rather than to depression. Another possibility is that the heavy workload is too recent for the impact to occur. Moreover, the negative effects of workload could be mitigated by the fact that workload is chosen rather than suffered (Lunau et al., 2014; Gerdenitsch, 2017). Indeed, 10% of respondents in our study were considering to pursue an academic career, which requires a greater personal investment. Moreover, the rate of residents reporting exposure to violence during their medical studies is a cause for concern (Hu et al., 2019) given the associated risks for health (post-traumatic stress disorder, anxiety, depression), as well as potential consequences for the curriculum (failed exams, dropping out of medical education). However, in our study, there was no specific association between exposure to violence and mental health. It is possible that the survey questions lacked precision by not exploring typical symptoms. Violence is a growing occupational risk in healthcare (Kannan et al., 2019; Sari et al., 2023). Further studies should explore more specifically the health and academic consequences for residents subjected to different types of violence in various contexts, such as hospitals, medical school, or private life.

In relation to university training conditions, a significant association was observed among one-third of the participants in the BASIL study between the perception of poor-quality training and a deterioration in mental health (low wellbeing, high level of psychological distress). Prior research has highlighted the imperative to enhance the quality of resident training, incorporating adaptations to new technologies (Mattar et al., 2013; Mann et al., 2020). Another study underscores the advantages of ensuring non-clinical training time, which may reduce burnout and enhance wellbeing (Stevens et al., 2020; Schlosser et al., 2023). Our results emphasize the importance of exploring the criteria influencing residents’ perception of training quality and understanding the mechanisms through which it correlates with their mental health. This original finding sheds light on the challenges faced by this specific population, as both emerging healthcare professionals in clinical settings and medical students still undergoing training.

Regarding personal determinants influencing residents’ mental health, various elements related to demographics, lifestyle habits and personality trait were identified. The duration of residency in France varies from 3 to 6 years, depending on the specialty. Being in the first part of residency (year 1 and 2) was associated with a lower level of wellbeing among BASIL participants, which is consistent with reported increases in depression in the literature (Mata et al., 2015). Younger age (i.e., below 30 years) is commonly associated with a lower sense of fulfillment (Shalaby et al., 2023). This association may be attributed to reduced resources and latitude at the beginning of residency, coupled with heightened adaptation demands during this transitional period. Approximately 60% of BASIL respondents had completed their externship outside the Lyon subdivision, requiring them to transition from their familiar environment to a new one, and to adapt accordingly. Being older (>29 years) was also associated with a lower level of wellbeing. This seemingly contradictory finding can be interpreted in different ways. Older residents may include those with children (4% of participants), who face greater challenges in balancing personal and professional responsibilities (Kocalevent et al., 2020). Additionally, they may be individuals preparing for a forthcoming period of adaptation between the end of their residency and the commencement of their professional career, which could be a potential source of anxiety and uncertainty.

In terms of lifestyle habits, residents most commonly reported unfavorable health behaviors included sleep problems, risky alcohol consumption, and sedentary behavior coupled with insufficient physical activity. Although no significant association was identified with their mental health at the time of the study, these behaviors remain a cause for concern for residents’ overall health, and is consistent with findings reported in other studies (Markwell and Wainer, 2009).

Concerning their personality traits, our study identified significant associations between certain personality traits and the mental health of residents. Specifically, the personality traits of “neuroticism” and “conscientiousness” were associated with a lower level of mental health. While the diversity of measures used in the literature complicates direct comparisons, these elements are crucial for comprehending and enhancing self-awareness and coping strategies (Louwen et al., 2023). Limited research has explored this subject, particularly among residents. Further studies are necessary to deepen our understanding of the connections between personality, environmental factors influencing residents’ coping strategies, and the subsequent impact on academic performance, wellbeing and overall health.

One of the most unexpected findings was that 17% of residents would not opt to undergo the same studies to become a doctor/dentist/pharmacist again. This result is particularly worrying, considering that mental health problems impact doctors of all categories and all ages in France. In a recent study involving 2,390 French teaching hospital physicians, almost 40% of respondents reported severe burnout, and 12% experienced job strain (Dres et al., 2023). A 2019 meta-analysis estimated the prevalence of burnout at 49% among French doctors, and at 52% among residents (Kansoun et al., 2019). Therefore, it is crucial to coordinate the prevention and promotion of residents’ mental health with that of the doctors who are tasked with supervising and collaborating with them during their studies.

### 4.2 Implications

The interweaving of environmental factors (hospital and university) and individual factors (lifestyle, personality) associated with residents’ mental health clearly illustrates the necessity of adopting an ecological approach to mental health promotion and prevention among residents.

According to French regulations, it is the responsibility of the employers to assess psychosocial risk factors for employee health and to implement necessary improvements. In this context, the University of Lyon and the Lyon University Hospitals consortium have collaborated to establish a prevention and health promotion plan specially tailored for residents.

Skills, motivation and personality traits are essential factors in shaping the development of excellent doctors (Khawar et al., 2022). The results concerning the different specialties of the residents need to be studied in greater depth, and merit a specific study. Working and academic conditions must be in line with this expectation. Emerging training opportunities, such as mentoring, provides avenues for improvement (Mann et al., 2020). In Lyon, the formal adoption of mentoring is limited (38.6% have a tutor and 35.2% have an equivalent mentor), despite its potential to enhance satisfaction with the chosen specialty (Enson et al., 2022). Exploring the impact of mentoring on resident mental health represents a significant area for intervention that warrants investigation. The mentoring approach holds particular promise in addressing the diverse challenges identified. Individualized support from a senior doctor for each resident has the potential to foster a gradual assumption of professional responsibility, accommodating individual personality and functioning. This, in turn, may reduce cognitive load and anxiety while expanding the autonomy and empowerment.

The individual determinants of mental health provide valuable insights into potential levers. Prevention initiatives targeting coping skills could contribute to enhancing residents’ individual experiences. In secondary and tertiary prevention, the implementation of specialized consultations have demonstrated the potential to improve health (Jacob et al., 2020). Healthcare professionals, at any stage in their training or career, can strengthen their professional and psychosocial skills, self-awareness and empowerment. Social support stands out as a pivotal factor at the intersection, positively contributing to primary prevention and mitigating various risk situations in healthcare work organizations (Blanch, 2016).

However, interventions must not be limited to individual determinants, but must also include professional and social determinants. Presently, knowledge available on the prevention and promotion of mental health in the workplace predominantly focuses on individual interventions, and it is necessary to develop interventions targeting modifications in the work environment (Tamminga et al., 2023; Waddell et al., 2023). Attention to workload and fostering a professional culture that values the right to disconnect emerged as crucial elements in the findings from BASIL. As per existing literature, work-family imbalance is a predictive factor for burnout symptoms among caregivers, doctors and residents. Conversely, maintaining a good work-life balance serves as a lever for reducing the risk of poor mental health and intention to leave the profession (Hämmig, 2018; Häusler et al., 2018; Kocalevent et al., 2020).

### 4.3 Study strengths and limitations

The participation rate of 46.7% among residents ensures the representativeness of the sample and the validity of the results. A comprehensive study examining mental health, working conditions, academic conditions and lifestyle habits provides an integrated perspective on health. Collaboration among residents, university and hospital management, the occupational health service, the university health service and multidisciplinary researchers in designing the study facilitated the development of a questionnaire that addressed the concerns of the various stakeholders. This collaboration also aided in relaying information about the study, encouraging resident participation.

Nevertheless, this study has several limitations. The results are specific to the Lyon subdivision and cannot be generalized to the 36,520 residents in France in 2021 (Castaing, 2021). There is a potential for selection bias, as residents more aware of mental health issues might have had a higher participation. However, efforts to minimize this bias were made through the survey’s dissemination across several channels and peer networks (resident unions). Likewise, the majority of the sample was female, which is usual in epidemiology surveys (Slauson-Blevins and Johnson, 2016). Although statistical analysis attempted to account for this over-representation, the lack of diversity of our sample is a limitation. Moreover, the use of the BFI-10 only allows the detection of personality dimensions based on the Big Five model. A technical issue with the online questionnaire prevented the collection of data on the agreeableness dimension of the BFI-10. According to the questionnaire’s validators, the factor structure of the BFI-10 is identical to that of the full BFI-Fr, with a very satisfactory rating for the factors “extraversion,” “conscientiousness,” “neuroticism” and “openness,” and an acceptable rating for “agreeableness” (Courtois et al., 2020). Finally, relying on self-reported questionnaires introduces inherent limitations.

## 5 Conclusion

The results of this survey confirm the concerns about the mental health status of health profession residents, and provide new data about the determinants influencing mental health, in particular within the work environment both at the hospital and the faculty. Additional determinants related to personal life, and lifestyle habits.

Efforts to prevent and promote mental health among residents at work must not be confined to providing individual support, and should be extended with evaluated actions implemented in the working and academic environments. These initiatives must be tailored to the complexity and local specificities of the evolving work organizations.

Collaboration among occupational health units, institutions (hospital and university), researchers, residents and senior professionals from hospitals and health universities is essential. As demonstrated in the BASIL barometer, this collaborative effort is crucial for formulating and endorsing ongoing actions to improve resident mental health at work.

## Data availability statement

The datasets presented in this article are not readily available because data analysis in progress. Requests to access the datasets should be directed to ludivine.nohales@chu-lyon.fr.

## Ethics statement

The studies involving humans were approved by the University of Lyon Research Ethics Committee (Comité d’éthique de la recherche de l’Université de Lyon, CER-UdL). The studies were conducted in accordance with the local legislation and institutional requirements. The participants provided their written informed consent to participate in this study.

## Author contributions

LN: Conceptualization, Formal analysis, Investigation, Methodology, Writing – original draft. EF: Data curation, Formal analysis, Investigation, Methodology, Writing – review & editing. SP: Conceptualization, Formal analysis, Investigation, Methodology, Writing – review & editing. CC: Investigation, Writing – review & editing. PL: Investigation, Writing – review & editing. JT: Formal analysis, Investigation, Methodology, Writing – review & editing. MW: Investigation, Writing – review & editing. BR: Conceptualization, Formal analysis, Investigation, Methodology, Writing – review & editing. J-BF: Conceptualization, Formal analysis, Investigation, Methodology, Writing – original draft.

## Consortium: Basil Study Group

Unions (SYREL MG, SAIHL and SIPHL), Senior scientists (J Haesebaert, F Haesebaert, S Mazza, P Michel, E Poulet, E Leaune, AM Schott), residents (L Lestienne, R Varnier, L Rodriguez-Borlado Salazar, V Arigault, X Balmelle), Sophie Granger (DAM).

## Supplementary material

Supplementary DATA SHEET 1 The health barometer of lyon subdivision residents.

Supplementary FIGURE 1 DAG: working conditions-psychological distress.

Supplementary FIGURE 2 DAG: academic conditions-psychological distress.

Supplementary FIGURE 3 DAG: academic conditions-wellbeing.

Supplementary FIGURE 4 DAG: working conditions-wellbeing.
