# Supplementary material for: Occupational, academic, and personal determinants of wellbeing and psychological distress in residents: results of a survey in Lyon, France
Source: Front Psychol. 2024 May 6;15:1347513. doi: 10.3389/fpsyg.2024.1347513 (PMC11103015; doi:10.3389/fpsyg.2024.1347513)
Supplement: Supplementary DATA SHEET 1 — The health barometer of lyon subdivision residents. [file Data_Sheet_1.PDF]

The Health Barometer of Lyon Subdivision Residents

(« Baromètre Santé des Internes de la Subdivision de Lyon », BASIL)

Socio-demographic data

- You are...
  - a woman
  - a man
  - other
  - I prefer not to answer.

- How old are you?

- Marital status: *Please select all that apply*
  - Single
  - In a relationship
  - Civil union
  - Married
  - Divorced
  - Widowed

- How many children do you have?

- Do you live alone?
  - Yes
  - No

- Were you in Lyon for your medical study before your residency?
  - Yes
  - No

- What year of residency are you in?
  - 1
  - 2
  - 3
  - 4
  - 5
  - 6
  - 7
  - 8
  - 9
  - 10

- What is your specialty? *N.B.: specialties with enrollments below 10 have been grouped together.*
  - Anesthesiology-Intensive Care / Emergency and Critical Care
  - Medical Biology

  - Obstetrics and Gynecology
  - Occupational Medicine / Public Health
  - General Medicine
  - Dentistry
  - Pediatrics
  - Pharmacy (excluding medical biology)
  - Psychiatry
  - Radiology / Nuclear Medicine / Genetics / Anatomopathology
  - Other surgical specialty
  - Other medical specialty

- Are you satisfied with your choice of specialty?
  - Not at all
  - Moderately
  - Completely

- Have you exercised a "right to change one's mind "?
  - Yes
  - No
  - Not applicable

- In which year did you take the National Ranking Exam (ECN)?

- Do you wish to pursue an academic career?
  - Yes
  - No
  - I don't know

Big Five Inventory BFI-10 (French items)

Working and academic conditions

- **Where are you** currently **working** as a resident (several choices possible)?
  - in the emergency department / resuscitation / intensive care unit (hospital / clinic)
  - in an obstetrics department (hospital/clinic)
  - in a surgical department (hospital/clinic)
  - in a psychiatric ward (hospital/clinic)
  - in a medicine department (hospital/clinic)
  - in outpatient care (hospital/clinic)
  - in the biology laboratory / pathology laboratory (hospital / clinic)
  - other: ...

- Overall, how would you rate your **well-being during your internship**?
  - Very poor
  - Poor
  - Fairly good
  - Good
  - Very good

- How would you rate the contribution of **your current internship** to your training?
    - Very poor
    - Poor
    - Fairly good
    - Good
    - Very good

- On average, **how many hours** do you work per week during your internship (including shifts and on-call duty)?

- Are you involved in night **shifts**?
  - yes
  - no
    - If yes, **how many** per month? *excluding half-night shifts: in this case 2 half-night shifts = 1 full shift*
      - < 1
      - 1
      - 2
      - 3
      - 4
      - 5
      - 6
      - 7
      - 8
      - 9
      - 10
      - > 10

    - Can you take **a safety break** (*total work interruption lasting at least 10 hours*) following a shift...?
      - Always
      - Almost always
      - Sometimes
      - Rarely
      - Never

- Do you have **on-call duty**?
  - yes
  - no
    - If yes, **how many times** per month?
      - < 1
      - 1
      - 2
      - 3
      - 4
      - 5
      - 6
      - 7
      - 8
      - 9
      - 10
      - >10

- Can you take a **safety break** *(total work interruption lasting at least 10 hours*) following an on-call duty...?
  - Always
  - Almost always
  - Sometimes
  - Rarely
  - Never

- Have you ever worked while you were sick and would have had to take a sick leave?
  - Never
  - Rarely
  - Sometimes
  - Often
  - Very often

- Are you currently pregnant or have you been **pregnant** during your residency?
  - yes
  - no
  - not applicable

  - If yes, are you currently experiencing or have you had **specific difficulties** in your work related to pregnancy? *Please select all applicable answers*
    - discrimination
    - difficulties related to on-call duties
    - other: ...

- On average, how much time per week do you dedicate to your work **outside your internship location**? ...
  - < 1h
  - 1h
  - 2h
  - 3h
  - 4h
  - 5h
  - 6h
  - 7h
  - 8h
  - 9h
  - 10h
  - >10h

- Is your weekly **half-day of university training available to you...**?
  - Always
  - Almost always
  - Sometimes
  - Rarely
  - Never

- Can you make use of your weekly **half-day for** of **personal training...**?
    - Always
    - Almost always
    - Sometimes
    - Rarely
    - Never

- Overall, how would you rate the contribution of your **postgraduate university courses** to your training?
  - Very Poor
  - Poor
  - Fairly good
  - Good
  - Very good

- Do you have a **tutor**?
  - yes
  - no
  - If yes: do you feel **confident** enough with your tutor to discuss any difficulties you may encounter?
    - yes
    - no

  - If not: are you in contact with a senior colleague whom you consider a "**mentor**" *(listening, advice, help in case of difficulty...)*?
    - yes
    - no
    - If not: would you like it to be the case?
      - yes
      - no

<u>Job Content Questionnaire (French items)</u>

- If you had to do it over, would you **choose to become a doctor/pharmacist/dentist** again?
  - Definitely not
  - Probably not
  - Not sure
  - Probably yes
  - Definitely yes

<u>Health</u>

- Currently, how would you rate your overall **health?**
  - Very good
  - Good
  - Neither good nor bad
  - Bad
  - Very bad

- Do you suffer from a chronic or long-term **health condition** affecting your quality of life?
    - Yes
    - No

- Currently, how would you rate your overall **quality of life**?
    - Very good
    - Good
    - Neither good nor bad
    - Bad
    - Very bad

Warwick-Edinburgh Mental Well-Being Scale (WEMWBS)

Kessler Psychological Distress Scale (K6)

- Have you ever been a victim of **violence** (verbal, physical, psychological or sexual) during your **studies**?
    - yes
    - no

    - If **yes,** what was the **nature of these acts of** violence? (*Multiple answers possible*)
        - verbal
        - psychological
        - physical
        - sexual

    - If **yes,** have you ever **confided in someone** about these acts of violence?
        - yes
        - no

    - If **no,** why?
        - fear of consequences
        - I don't know who to talk to
        - I didn't see the point.
        - other reason

- During the last **12 months**, have you seen or consulted a healthcare professional regarding your **emotional or mental health**?
    - yes
    - no

    - If yes, **whom** did you consult?
        - General practitioner
        - Psychiatrist
        - Psychologist
        - Nurse
        - Social worker or counselor
        - Other: ...

- During the **last 12 months**, have you taken any of the following medications?

	every day	several times a week	a few times a month	Less frequently	never
Sleeping pills					
Anxiolytics					
Antidepressants					
Neuroleptics					
Psychostimulants					

  - If at least one box is checked: were any of these drugs **self-medicated**?
    - Yes
    - No

      - If yes: **why**? ...

Lifestyle

- Do you think your diet is **balanced**?
  - Absolutely = 10 9 8 7 6 5 4 3 2 1 0 = Not at all

- On the days when you're **on internship**, would you say you have **enough time** to eat?
  - Never
  - Rarely
  - Sometimes
  - Often
  - Very often

- On average, how many hours **per week do** you dedicate to **intense physical activity** (sports or other)? ...

- On average, how many hours **per week do** you dedicate to **moderate physical activity** (e.g. walking)? ...

- On average, how many hours do you spend **sitting each day** (considering all situations: work, transportation, at home, leisure...)? ...

- Please estimate the current **severity** (last month) of your sleep difficulties

Difficulty falling asleep
None
Slight
Moderate
Very
Extremely

Difficulty staying asleep
None
Slight
Moderate

Very
Extremely

Problems waking up too early in the morning
None
Slight
Moderate
Very
Extremely

- **To what extent are you satisfied / dissatisfied** with your current sleep?

	Not at all	Slightly	Moderately	Very	Extremely
To what extent do you consider that your sleep difficulties disrupt your daily functioning?					
To what extent do you consider that your sleep difficulties are apparent to others in terms of deterioration in the quality of your life?					
To what extent are you worried / concerned about your sleep difficulties?					

- On average, how many <u>hours per day do</u> you spend in front of a **screen** (computer, tablet, smartphone, ...) as **part of your professional activities**? ...

- On average, how many <u>hours per day do</u> you spend in front of a **screen** (computer, tablet, smartphone, ...) **outside of your professional activities** (transportation, at home, leisure, ...)? ...

- When I go on weekends / on vacation, it is possible for me not to check my **work emails.**
    - Absolutely
    - Not all the time
    - Never

<u>Consumption of psychoactive substances</u>

- Do you **smoke tobacco** ?
    - I have never smoked
    - I used to smoke but I don’t currently
    - I smoke occasionally (not every day)
    - I smoke tobacco daily (at least 1 cigarette per day)

- **If you** currently **smoke** tobacco, do you plan to quit?
    - Yes, in the coming weeks
    - Yes, in the more distant future
    - No
    - I haven't considered it

- How often do you consume **alcoholic** beverages?

- Never
- Once a month or less
- 2 to 4 times a month
- 2 to 3 times a week
- 3 or more times per week

- How many standard drinks do you have on a **typical day when you consume alcohol**?
  - 1 or 2
  - 3 or 4
  - 5 or 6
  - 7 or 9
  - 10 or more
- **On a single occasion, how often do** you drink six or more standard drinks?
  - Never
  - Less than once a month
  - Once a month
  - Once a week
  - Almost every day

- In the **last 30 days**, how often have you used **cannabis**?
  - Never
  - 1 to 2 times
  - 3 to 9 times
  - 10 times or more
  - Every day

- Have you used any of the following products in the **last 12 months**?

	No	1 to 2 times	3 to 5 times	6 to 9 times	10 times or more
Hallucinogenic mushrooms					
LSD					
Inhalants (poppers, glue, solvents)					
Nitrous oxide					
Ecstasy, MDMA					
Amphetamines, speed					
Cocaine					
Crack					
Heroin					
Other drugs (excluding cannabis): specify ...					
