# Supplementary figures and images for: Occupational, academic, and personal determinants of wellbeing and psychological distress in residents: results of a survey in Lyon, France
Source: Front Psychol. 2024 May 6;15:1347513. doi: 10.3389/fpsyg.2024.1347513 (PMC11103015; doi:10.3389/fpsyg.2024.1347513)

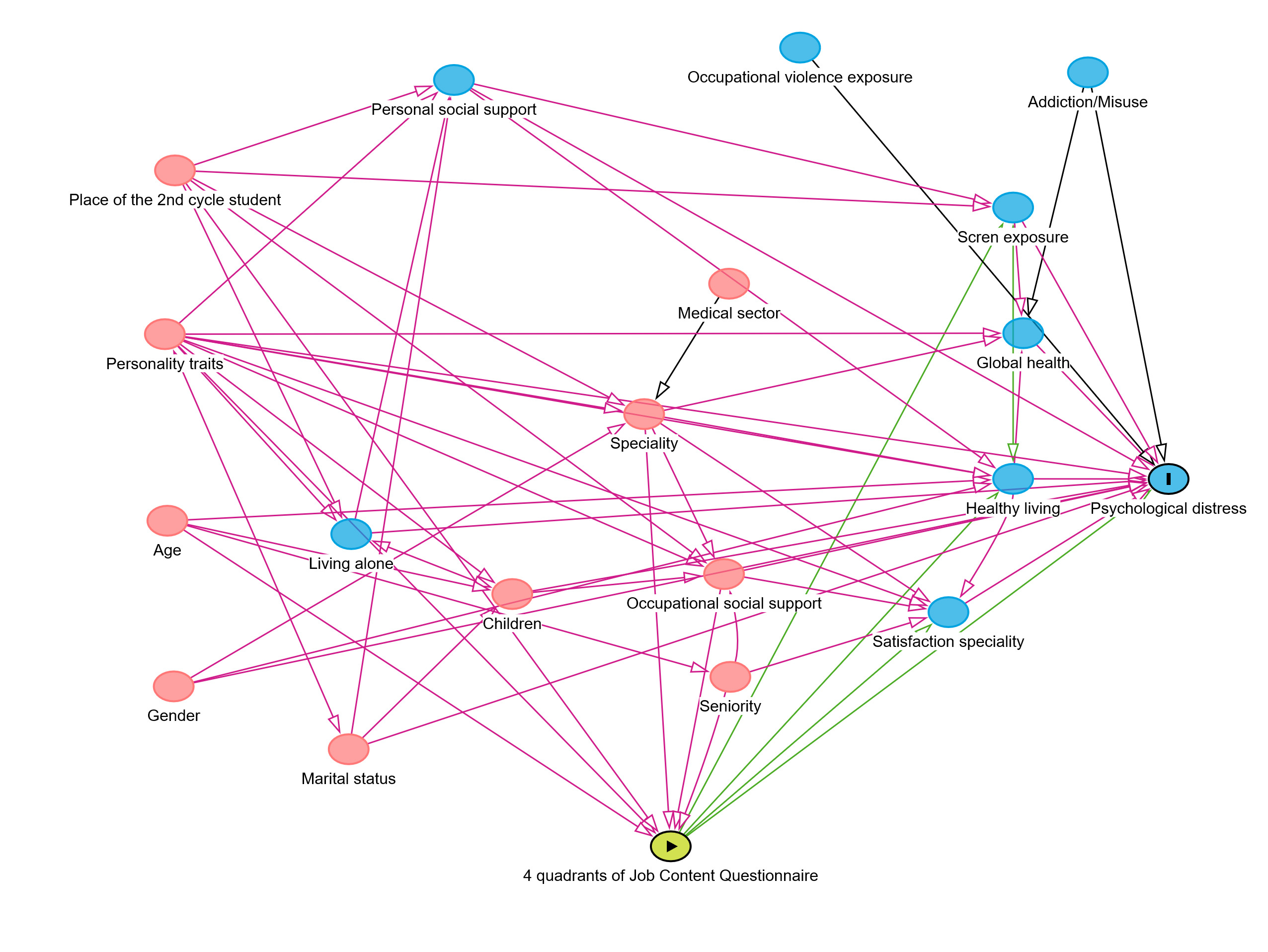

Supplement: Supplementary FIGURE 1 — DAG: working conditions-psychological distress. [file Image_1.JPEG]

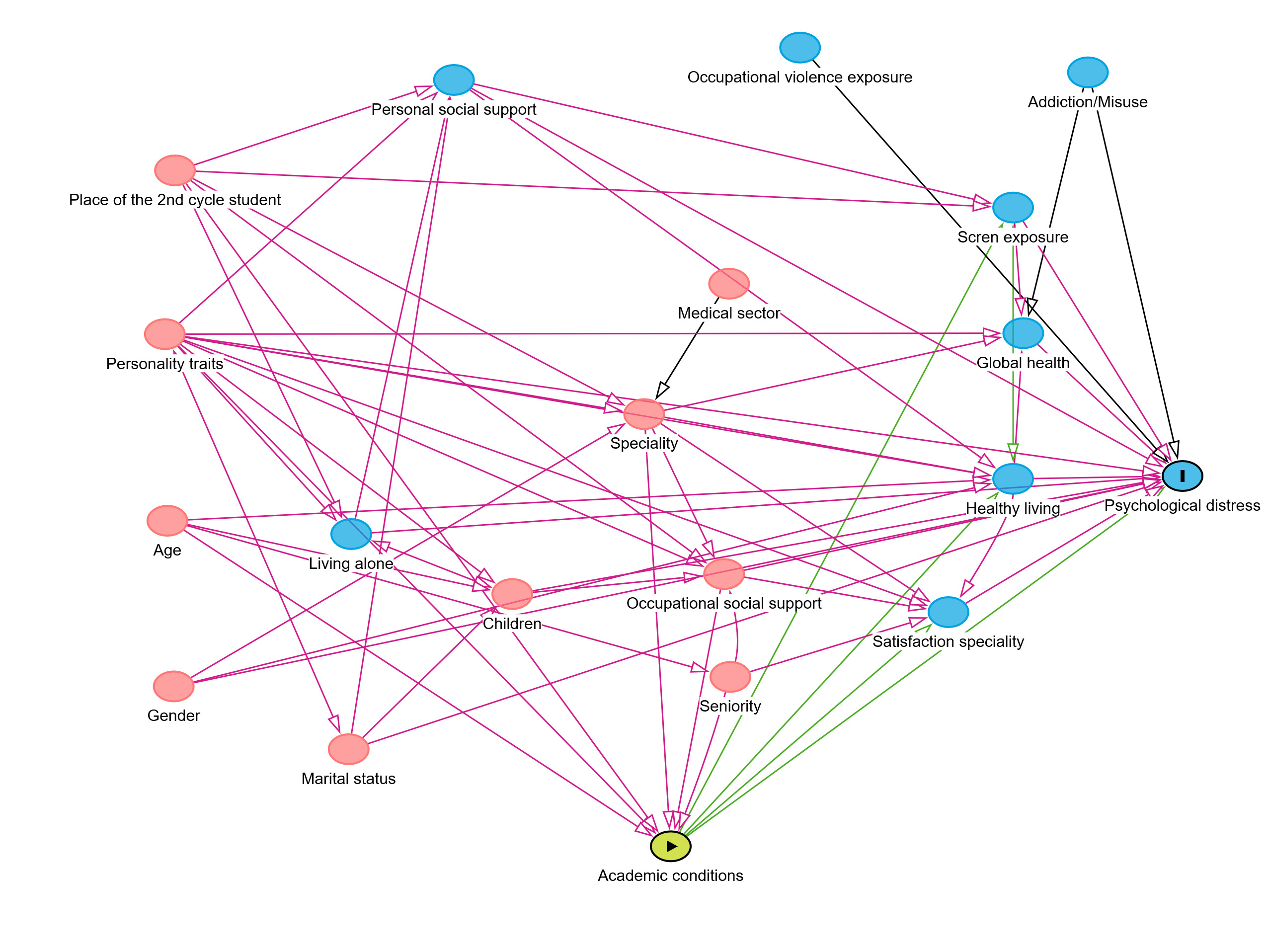

Supplement: Supplementary FIGURE 2 — DAG: academic conditions-psychological distress. [file Image_2.JPEG]

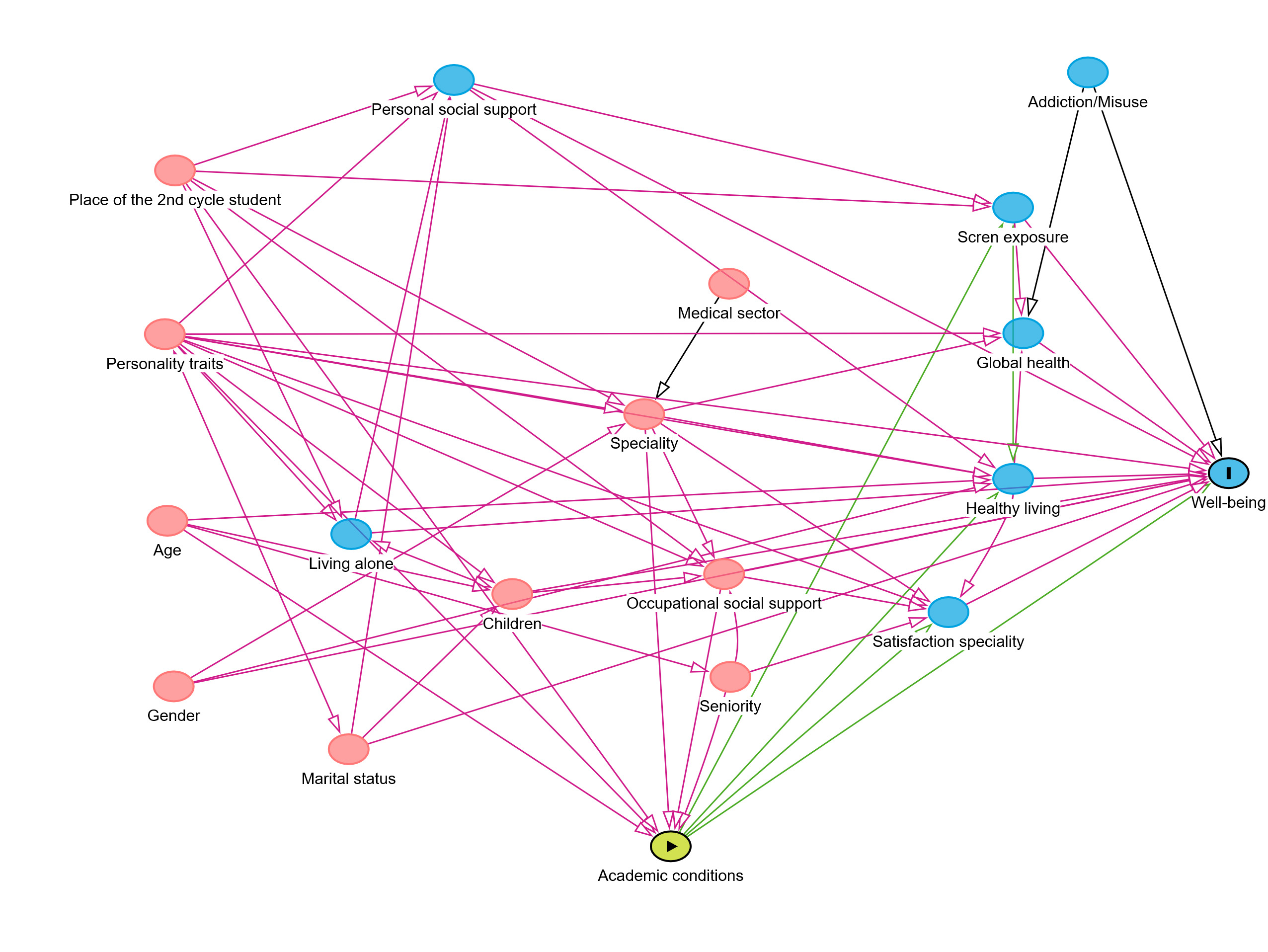

Supplement: Supplementary FIGURE 3 — DAG: academic conditions-wellbeing. [file Image_3.JPEG]

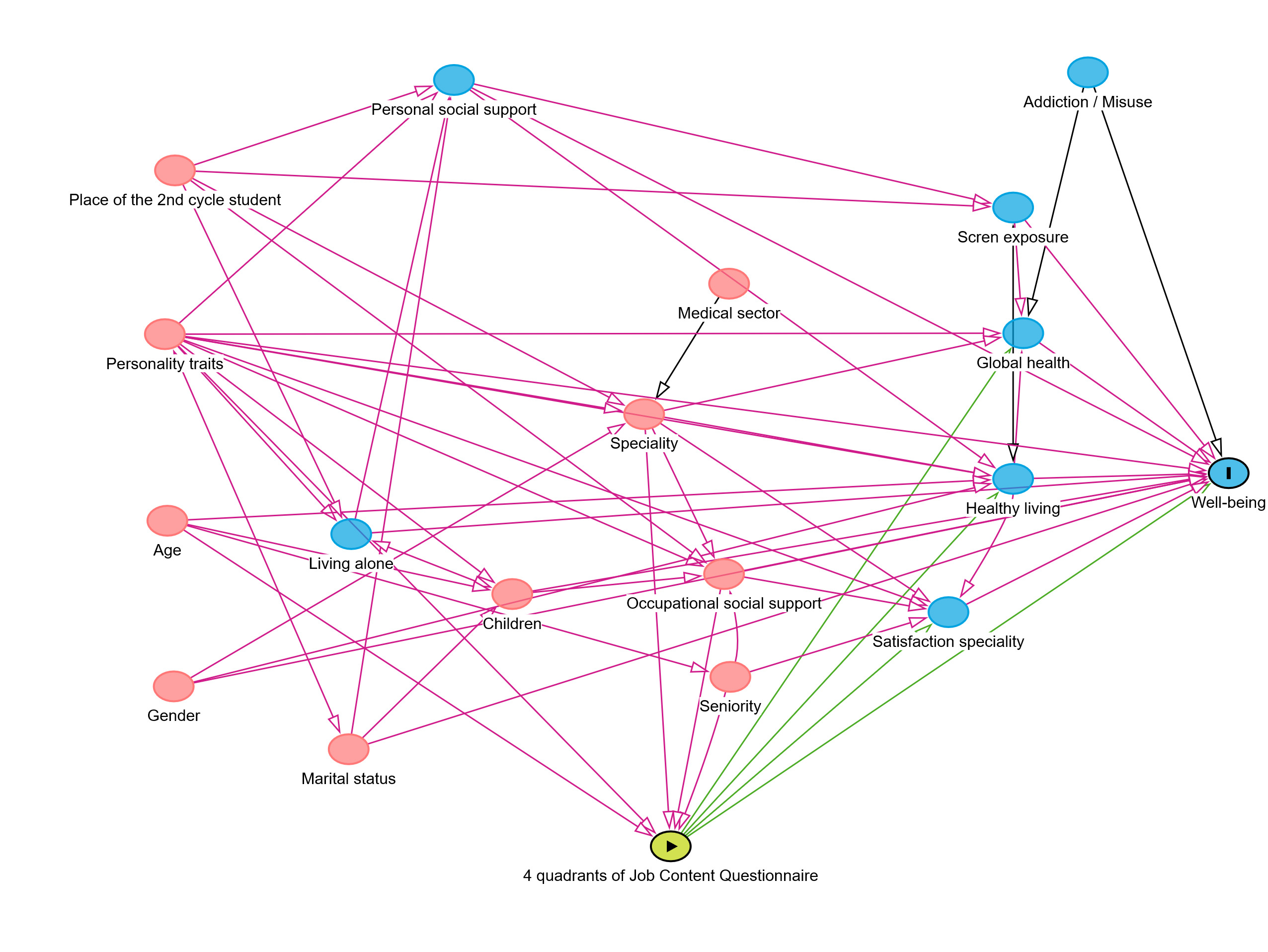

Supplement: Supplementary FIGURE 4 — DAG: working conditions-wellbeing. [file Image_4.JPEG]
